# Functional characterization of TSPAN7 as a novel indicator for immunotherapy in glioma

**DOI:** 10.3389/fimmu.2023.1105489

**Published:** 2023-02-09

**Authors:** Long Chen, Hongwei Liu, Yanwen Li, Xuelei Lin, Shunjin Xia, Siyi Wanggou, Xuejun Li

**Affiliations:** ^1^ Department of Neurosurgery, Xiangya Hospital, Central South University, Changsha, China; ^2^ Hunan International Scientific and Technological Cooperation Base of Brain Tumor Research, Xiangya Hospital, Central South University, Changsha, Hunan, China; ^3^ Xiangya School of Medicine, Central South University, Changsha, China

**Keywords:** glioma, single-cell RNA sequencing, malignant progression, immune infiltration, immunotherapy

## Abstract

Glioma is the most common primary malignant tumor of the central nervous system in clinical practice. Most adult diffuse gliomas have poor efficacy after standard treatment, especially glioblastoma. With the in-depth understanding of brain immune microenvironment, immunotherapy as a new treatment has attracted much attention. In this study, through analyzing a large number of glioma cohorts, we reported that TSPAN7, a member of the tetraspanin family, decreased in high-grade gliomas and low expression was associated with poor prognosis in glioma patients. Meanwhile, the expression pattern of TSPAN7 was verified in glioma clinical samples and glioma cell lines by qPCR, Western Blotting and immunofluorescence. In addition, functional enrichment analysis showed that cell proliferation, EMT, angiogenesis, DNA repair and MAPK signaling pathways were activated in the TSPAN7 lower expression subgroup. Lentiviral plasmids were used to overexpress TSPAN7 in U87 and LN229 glioma cell lines to explore the anti-tumor role of TSPAN7 in glioma. Moreover, by analyzing the relationship between TSPAN7 expression and immune cell infiltration in multiple datasets, we found that TSPAN7 was significantly negatively correlated with the immune infiltration of tumor-related macrophages, especially M2-type macrophages. Further analysis of immune checkpoints showed that, the expression level of TSPAN7 was negatively correlated with the expression of PD-1, PD-L1 and CTLA-4. Using an independent anti-PD-1 immunotherapy cohorts of GBM, we demonstrated that TSPAN7 expression may had a synergistic effect with PD-L1 on the response to immunotherapy. Based on the above findings, we speculate that TSPAN7 can serve as a biomarker for prognosis and a potential immunotherapy target in glioma patients.

## Introduction

Gliomas are the most common malignant tumors of the central nervous system (CNS) in adults, including WHO grades I-IV (1), among which glioblastoma (GBM) has the worst prognosis (2, 3). The current standard treatments include surgical resection followed by radiotherapy, chemotherapy, immunotherapy and tumor treatment field. However, the prognosis of glioma is still unsatisfactory, which root cause lies in the invasive growth characteristics of glioma itself and the existence of inter- and intra-tumor heterogeneity (4, 5). For further understanding the molecular mechanisms behind the manifestation and progression, new prognostic biomarkers and effective targets for glioma still need to be identified.

Tumor immune microenvironment (TIME) plays a vital role in anti-tumor immune response. The interactions between tumor cells and immune cells in the TIME affect the progression of glioma. Immense amounts of concrete research have confirmed that tumor cells can modify the TIME during the progression of glioma, making it inclined to be an immunosuppressive state. For example, tumor cells would secrete inhibitory cytokines IL-10 and TGF-β, while overexpressing CTLA-4L and PD-1L to inhibit activated cytotoxic T cells (CTLs) (6, 7). So, it is urgent to carry out more efforts for exploring effective therapeutic regimens targeting tumor microenvironment. Recent years, PDCD1 (PD-1), CD274 (PD-L1) and CTLA4 have been confirmed to act as immune checkpoints and can prevent the immune system from killing cancer cells by inhibiting autoimmunity (8, 9). Therefore, the application of anti-CTLA4, anti-PDCD1 (PD-1) and anti-CD274 (PD-L1) agents is becoming a promising therapeutic option in several types of cancers. However, only a minority of glioma patients would benefit from immunotherapy, which demonstrating some unknown factors in the TIME may synergize with immune checkpoint blockades above influencing therapeutic outcome. To address these sparsity of knowledge, large-scale sequence data including bulk RNA sequencing and single-cell RNA sequencing (RNA-seq) are employed for computational analysis to identify novel biomarkers for glioma (10). Integrated high-throughput analysis and experiment validation enables researchers to elucidate new molecular mechanisms involved in immune response.

TSPAN7, also known as TM4SF2, located on chromosome X p11.4, is a protein coding gene. The protein encoded by this gene is a member of the tetraspanin family. Most of these members are cell-surface proteins that are characterized by the presence of four hydrophobic domains (11). The proteins mediate signal transduction events that play a role in the regulation of cell development, activation, growth and motility (12). Notably, this encoded cell surface glycoprotein has been demonstrated to exert a vital role in the control of neurite outgrowth (13). TSPAN7 was first introduced in T-cell acute lymphoblastic leukemia (ALL) and widely expressed in non-hematopoietic cells, of which the highest expression is in brain tissue (14, 15). Previous studies about TSPAN7 have been explored in different tumors. In oral tongue squamous cell carcinoma, different methylation status of TSPAN7 is considered to be a useful predictor of clinical prognosis within oral cancer patients (16). Cheong et al. found that in multiple myeloma (MM), TSPAN7 expression is associated with better outcomes (17). Besides, Wang et al. found that elevated TSPAN7 promotes lung cancer cells proliferation and migration *via* EMT (18). In additional, low expression of TSPAN7 was significantly correlated with poor prognosis in bladder cancer and clear cell renal cell carcinoma (19, 20). Overall, these findings support the speculation that TSPAN7 might be involved in the regulation of tumor progression of gliomas.

Here, we used several public online databases, including The Cancer Genome Atlas (TCGA), Chinese Glioma Genome Atlas (CGGA) and Gene Expression Omnibus (GEO), to analyze the relationship among the expression of TSPAN7, clinicopathological features, prognosis and immune microenvironment in glioma. Moreover, we collected glioma samples from Xiangya Hospital to confirm what we found from sequencing data *via* real-time quantitative polymerase chain reaction (qPCR), Western Blotting and immunofluorescence (IF). Based on these results, we speculate that TSPAN7 is a potential prognostic biomarker and may be developed as a clinical therapeutic target for glioma.

## Materials And Methods

### Public Data and Clinical Specimens Collection

In this study, the TCGA pan-cancer RNA-seq including lower grade glioma (LGG) and GBM with their clinical data were downloaded from the University of California, Santa Cruz (UCSC) Xena through the online data website (https://xenabrowser.net/). Two batches of CGGA RNA-seq including CGGA_693 and CGGA_325 with their clinical data were obtained from the CGGA website (http://www.cgga.org.cn/). All the RNA-seq expression data were log2 TPM transformed. In addition, microarrays of glioma cohorts (GSE16011, GSE108474, GSE43289) were also obtained from GEO database. All the microarray data were log normalized. Single-cell RNA-Seq data acquired from GSE84465, which including four primary GBM and 3589 cells in total have been standardized through R package Seurat. Processed spatial transcriptomic data were downloaded from R package SPATAData (https://github.com/theMILOlab/SPATAData) and analyzed by R package SPATA2. Besides, one GBM immunotherapy-related cohort including 14 samples with pre-therapy and post-therapy state was obtained from the SRA database (PRJNA482620), whose raw sequencing data were mapped to the hg38 reference genome through HISAT2 and log_2_TPM transformed. Finally, normal brain tissue (n=9) and glioma samples (n=27) were collected from the neurosurgery department of Xiangya Hospital of Central South University to detect the expression of TSPAN7.

### Single cell RNA-seq process

We normalized the single cell data by the function SCTransform in Seurat. Then, we selected the top 2000 features to perform dimensionality reduction, which was projected into t-distributed Stochastic Neighbor Embedding (t-SNE). Finally, the same marker profiles of the cell type reported in the original paper were compared with our current study to define the type of each cell. In order to identify subpopulations with bulk sample phenotype, we used Rpackage Scissor algorithm (21) to classify each cell into Scissor+ cells and Scissor- cells by combining TCGA survival outcomes. Scissor+ cells also marked group 1 were associated with worse survival while Scissor- cells also marked group 2 were associated with good survival.

### Survival Analysis

According to median expression level of TSPAN7, patients were divided into TSPNA7 high expression subgroup and low expression subgroup for purpose of assessing prognostic value. Kaplan Meier plotter was mainly used to describe survival distribution. Logarithmic rank test was used to evaluate the statistical differences between subgroups. Multi-Cox Regression by R package ezcox were applied to analyze the association between clinical factors and overall survival.

### Differential Gene Identification and Enrichment Analysis

Differential expression genes (DEG) were identified by using R package limma. False discovery rate (FDR) <0.05 and |log_2_FC (fold change)| >2.0 were used to define the cut-off value among DEGs. The R package clusterProfiler (22) was used to conduct Gene Ontology (GO) enrichment analysis on the up and down regulated DEG.

### Subtypes identification and gene set enrichment analysis

Bulk transcriptome subtypes of glioma were identified by R package ssgsea.GBM.classification (23) which classified glioma into proneural (PN), mesenchymal (MES) and classical (CL) subtype. This method outputs an enrichment score and a P-value of each of the three subtypes in a sample. To specify a subtype for each sample, the subtype with the lowest p-value were selected. For single cell subtypes of GBM (MES-like, AC-like, OPC-like, and APC-like) were identified using the same method from previous study (24). A total of 50 hallmark gene sets were obtained from the molecular signature database (MSigDB, http://software.broadinstitute.org/gsea/msigdb). Single sample gene set enrichment analysis (ssGSEA) method was used to analyze the enrichment scores of each hallmark gene set for each sample through R package GSVA.

### Immune infiltration analysis

The relationship between tumor infiltrating immune cells and gene expression level can be analyzed using CIBERSORT deconvolution algorithm, which mainly uses vector regression model to deconvolute cell types. 20 checkpoints with therapeutic potential (25) were collected to detect their correlations with TSPAN7.

### qPCR

Total RNA was extracted from clinical glioma samples according to TRIzol (Accurate Biology, China, AG21101) and RNA extraction kit (Thermo Scientific, K0731). And then, the total RNA was packaged and stored in a refrigerator at -80°C. Before the experiment, the total RNA was taken for amplification to obtain cDNA and qPCR. The PCR reaction conditions were: 95°C 15 min, 95°C 15 s, 60°C; 1 min, and 40 cycles. The amplification efficiency and specificity of the designed primers were analyzed by experimental data, and the specificity of the primers was analyzed by dissolution curve. GenBank queries the gene sequence and designs the primer sequence as follows: TSPAN7: (forward) 5 ‘- STATCCTTCGTCTTCGGATC-3’, (reverse) 5 ‘- CATACAGTTTCAGCATCGG-3’.

### Western Blot

The clinical glioma tissue samples and cell line samples were collected respectively. The total protein was extracted with RIPA lysate, the protein concentration was detected using BCA method (Bicinchonininc Acid), and the sample loading was calculated. Boil at 100°C for 10 min for protein denaturation, and store at -80°C for next use. Using 80v low voltage for electrophoresis at 12% SDS-PAGE. Until the minimum molecular weight band in marker reaches to the bottom of gel, transfer the target protein onto the PVDF membrane under the condition of 200 mA constant current for 80min. The membrane was blocked with QuickBlock™ Western Block Solution (Beyotime, P0252-100 ml) for 15min, washed it with PBST (phase buffer solution with 0.05% Tween 20) at room temperature, incubated the primary antibody (TSPAN7, abcam, ab211870) at 4°C overnight, then PBST washing, and incubated the second antibody at room temperature for 1.5 h. Using the Efficient Chemistry Kit (Genview, GE2301-100ML) in X-ray films (Bio-Rad) to observe the target band and internal reference band. Use ImageJ software to read the band signal intensity and analyze the relative protein expression.

### Immunofluorescent staining

The glioma samples taken from the surgery were collected for OCT embedding, stored at -80℃, sliced with a microtome (thickness: 10um). Slices were placed in the precooled PBS to dissolve the OCT glue, fixed with precooled acetone for 10min, incubated with 0.5% Triton X-100 for 15min, and blocked for 1h at room temperature with PBSTx+10% HINGS. Primary antibody (TSPAN7, Proteintech, 18695-1-AP) incubate at 4°C overnight, PBS washing, following Alexa Fluor-488 secondary antibody incubated at room temperature and away from light for 1h. DAPI was added to stain the nucleus and incubated in dark for 10 minutes. Anti-Fade Mounting Medium (Sangon Biotech, E675011) was used in the darkroom for sealing slices, dry at 37°C, and images was taken using fluorescence microscope.

### Flow Cytometry

GBM cells were harvested using trypsin without EDTA and then suspended by 500 μL 1×Binding buffer. Taking out 100ul to make the total number of cells 1 × 10^5^cell, add 5ul Annexin V-PE and 7ul 7-AAD (Meilunbio, MA0429, China). Incubated at room temperature in dark for 15min, add 400ul 1 × Binding buffer, after mixing, use FACS Calibur flow cytometer (BD Biosciences) to detect (within 1h). The results were analyzed with FlowJo10.4 software.

### Construct over-expression plasmid

TSPAN7 overexpression lentivirus was constructed in Hanbio Biotechnology (Shanghai, China), and the transfection protocol followed the manufacturer’s instructions. The sequence of TSPAN7 was obtained from NCBI, subcloned into lentivirus vector to overexpress TSPAN7, and the empty vector was used as negative control. The cells were transfected with lentivirus for 48-72 hours, and then the transfection efficiency of TSPAN7 overexpression was observed and analyzed using fluorescence microscope.

### Statistical Analysis

R version 4.1.0 (http://www.rproject.org/), GraphPad Prism 8 and Adobe Illustrator software were used to conduct bioinformatic statistical analyses and graph generation. Continuous variables fitting a normal distribution between binary groups were compared using a T-test, otherwise Wilcoxon-test. Correlations between variables were explored using Pearson or Spearman coefficients. All statistical significance were considered two-tailed tests and p < 0.05.

## Results

### 1 Clinicopathological Characteristics of TSPAN7 in Gliomas

**Figure 1:**
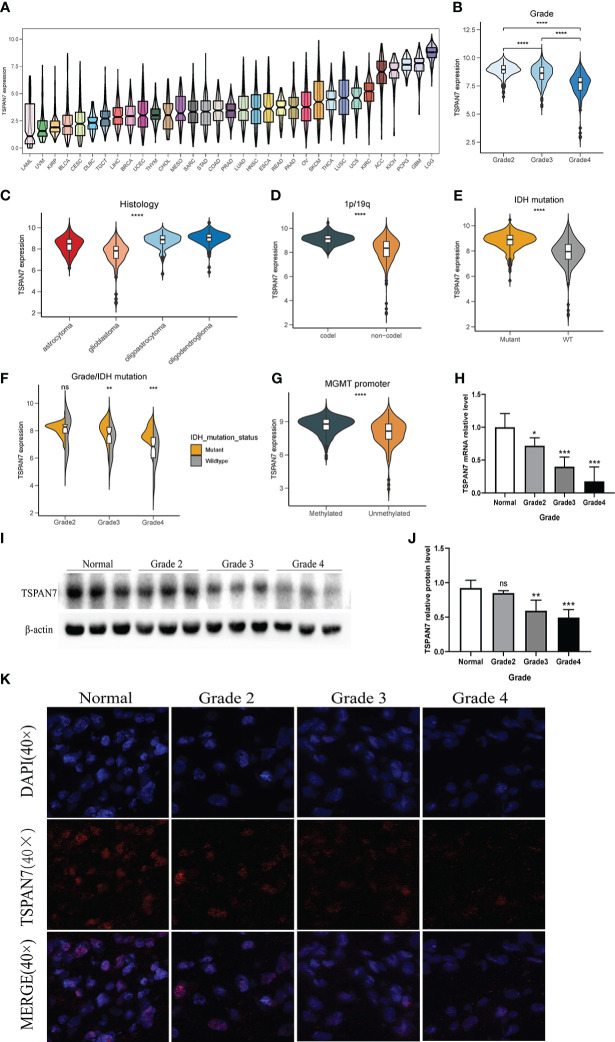
Clinical and molecular characteristics of TSPAN7 in gliomas. **(A)** The expression of TSPAN7 in multiple cancer from TCGA dataset. LAML, acute myeloid leukemia; UVM, Uveal Melanoma; KIRP, kidney renal papillary cell carcinoma; BLCA, bladder urothelial carcinoma; CESC, cervical squamous cell carcinoma and endocervical adenocarcinoma; DLBC, lymphoid neoplasm diffuse large B-cell lymphoma; TGCT, testicular germ cell tumors; LIHC, liver hepatocellular carcinoma; BRCA, breast invasive carcinoma; UCEC, uterine corpus endometrial carcinoma; THYM, thymoma; CHOL, cholangiocarcinoma; MESO, Mesothelioma; SARC, sarcoma; STAD, stomach adenocarcinoma; COAD, colon adenocarcinoma; PRAD, prostate adenocarcinoma; LUAD, lung adenocarcinoma; HNSC, head and neck squamous cell carcinoma; ESCA, esophageal carcinoma; READ, rectum adenocarcinoma; PAAD, pancreatic adenocarcinoma; OV, ovarian serous cystadenocarcinoma; SKCM, skin cutaneous melanoma; THCA, thyroid carcinoma; LUSC, lung squamous cell carcinoma; UCS, uterine carcinosarcoma; KIRC, kidney renal clear cell carcinoma; ACC, adrenocortical carcinoma; KICH, kidney chromophobe; PCPG, pheochromocytoma and paraganglioma; GBM, glioblastoma multiforme; LGG, brain lower grade glioma. **B, C**The expression of TSPAN7 stratified by WHO pathological grades and types. **(D, E)** The expression of TSPAN7 in 1p19q codeletion and non-codeletion glioma and IDH mutant and wild-type status. **(F)** The expression of TSPAN7 in IDH mutation status combined with different pathological grades. **(G)** The expression of TSPAN7 stratified by MGMT promoter methylation status. **(H)** The mRNA relative level of TSPAN7 in clinical glioma samples according to different WHO pathological grades. **(I, J)** Representative images of Western Blot for TSPAN7 in normal brain tissue and different pathological grades. **(K)** Representative images of IF staining for TSPAN7 in normal brain tissue and different WHO pathological grades of glioma. *p<.05, **p <.01, ***p <.001, ****p <.0001, ns: no statistics.

To explore the expression pattern of TSPAN7 in glioma, first we analyzed the expression of TSPAN7 in pan-cancer. The result showed that the TSPAN7 expression in LGG and GBM was significantly higher than that in other system cancers, and the expression level in LGG was significantly higher than that in GBM (**Figure 1A**). In TCGA, CGGA_325 and CGGA_693 datasets, we found that with the increase of tumor grade, TSPAN7 expression decreased gradually (**Figure 1B**, **Supplementary Figures S1A, B**). Similarly, according to the classification of different histological types, TSPAN7 showed the lowest expression in GBM, followed by astrocytoma (**Figure 1C**). The same results could be found in CGGA_325 and CGGA_693 (**Supplementary Figures S1C, D**). These results suggested that with the increase of malignance of glioma, the expression of TSPAN7 shows a gradually decreasing trend. As IDH mutation and 1p/19q deletion status are the dominant molecular marker for glioma patients, we investigate the relationship between them and TSPAN7 expression in TCGA and CGGA. The results showed that the expression of TSPAN7 in 1p/19q co-deletion group was significantly higher than that in 1p/19q non-codeletion group (**Figure 1D**, **Supplementary Figures S1E, F**). Meanwhile, TSPAN7 expression in IDH mutation was also significantly higher than that in IDH wild type, especially in grade 3 (**Figures 1E, F**, **Supplementary Figures S1G–J**). Additionally, in both TCGA and CGGA datasets, it was found that the expression level of TSPAN7 was higher in the MGMT methylation subgroup compared with MGMT non-methylation subgroup suggesting that TSPAN7 high-expressed patients may be sensitive to chemotherapy. (**Figure 1G**, **Supplementary Figures S1K, L**).

In order to verify the above bioinformatics findings, we collected glioma tissues undergoing surgical treatment in the neurosurgery department of Xiangya Hospital and detected the expression of TSPAN7 in glioma samples and normal brain tissue by qPCR, Western Blot and immunohistochemistry. The clinicopathological characteristics of the patients are shown in the **Supplementary Table S1**. The results suggested that compared with LGG, TSPAN7 mRNA and protein expression levels were lower in GBM (**Figures 1H–K**).

### 2 Low TSPAN7 expression level shows a subtype preference in GBM

**Figure 2:**
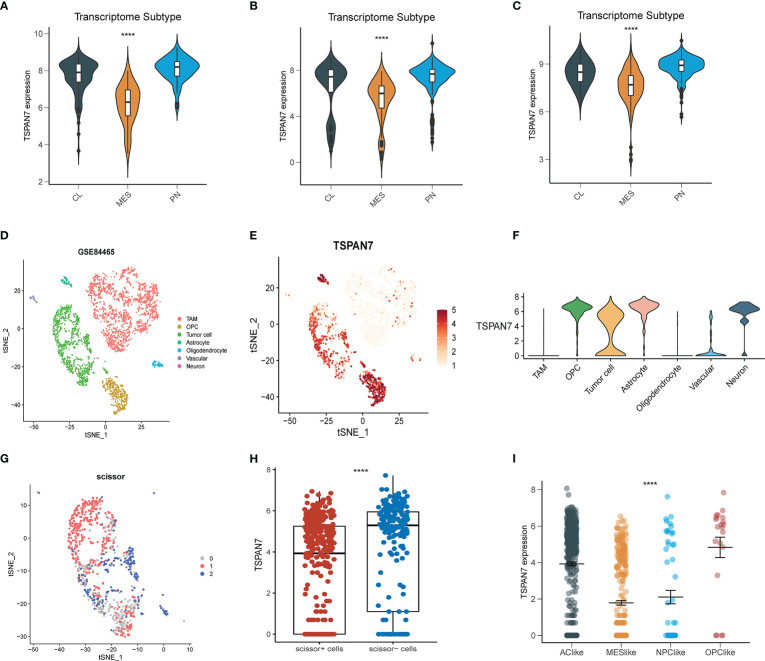
The expression of TSPAN7 in different glioma subtypes **(A-C)** TSPAN7 expression level in different molecular subtypes in CGGA_325, CGGA_691 and TCGA datasets based on bulk expression. **(D-E)** The cells were categorized into seven clusters and scatter plots represent TSPAN7 expression distribution in seven clusters. **(F)** The expression of TSPAN7 in seven clusters. **(G)** single cell was divided into Scissor+ cell or Scissor- cell 3 groups according to the prognosis by scissor algorithm. **(H)** The expression of TSPAN7 in Scissor+ cell and Scissor- cell. **(I)** Tumor cells were extracted and categorized into four different cell clusters based on scRNA expression level. ****p <.0001.

The molecular subtypes of glioma are of great significance to the prognosis of glioma patients. To further elucidate the expression pattern of TSPAN7 distributed in subtypes, transcriptomic classification was performed at bulk level and single cell level. In bulk level, TSPAN7 was highly expressed in Pro-neutral (PN) subtype, while low expressed in Mesenchymal (MES) subtype in TCGA, same as in CGGA_325 and CGGA_693 datasets (**Figures 2A–C**). Because of heterogeneity in tumor microenvironment, we further investigated whether TSPAN7 was specifically expressed in glial lineage. Single cell RNA-seq data of GSE84465 identified 7 cell clusters including tumor associated macrophages (TAM), oligodendrocyte progenitor cells (OPC), tumor cell, astrocyte, oligodendrocyte, vascular and neuron (**Figure 2D**). The expression of marker genes of each cluster in single cell sequencing data was shown in **Supplementary Figures S2A, B**. Of note, TSPAN7 was mainly expressed in glial cells in which the expression in tumor cell was lower than normal cell such as OPC and astrocyte consistent with our previous findings (**Figures 2E, F**). Besides, single cell was divided into Scissor+ cell or Scissor- cell by scissor algorithm (**Figure 2G**), and the expression of TSPAN7 was higher in Scissor- cells indicating its association with better prognosis again (**Figure 2H**).

As Neftel et al. showed there existing four subtypes in GBM microenvironment namely neural-progenitor-like (NPC-like), oligodendrocyte-progenitor like (OPC-like), astrocyte-like (AC-like), and mesenchymal-like (MES-like), the MES subtype at bulk level corresponds to the MES-like subtype at single cell level. The results showed that the expression of TSPAN7 was the lowest in MES-like subtype, which was consistent with the expression pattern at bulk level (**Figure 2I**). Moreover, we also analyzed the expression pattern of TSPAN7 in the spatial transcriptome (26), and found that MES-like scores were also relatively lower in areas with relatively higher TSPAN7 expression, and vice versa (**Supplementary Figures S3A–C**). These results suggested that TSPAN7 was more likely expressed in MES subtype at both bulk and single cell level and may play a pivotal role in the transformation of GBM subtypes.

### 3 High expression of TSPAN7 infers a better prognosis for glioma

**Figure 3:**
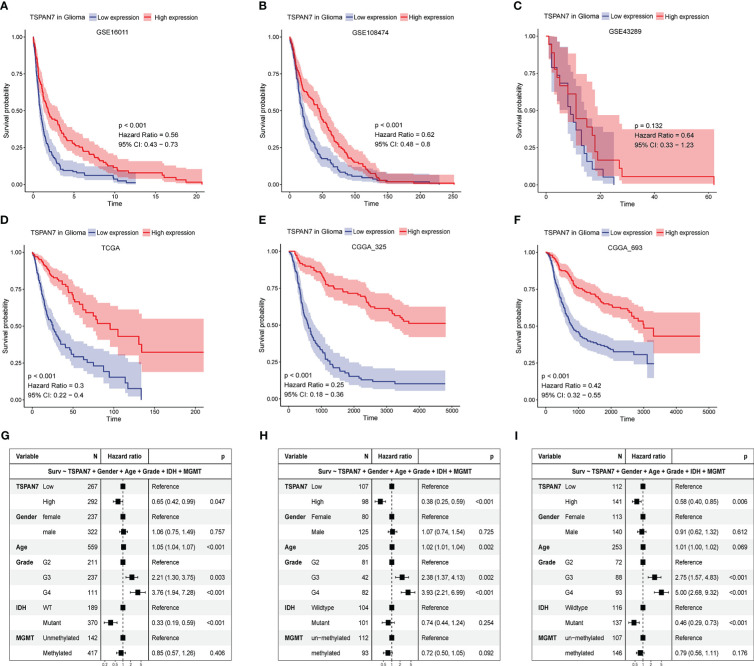
The relationship between TSPAN7 expression and survival for glioma. **(A-F)** Kaplan–Meier plots of TSPAN7 in **(A)** GSE16011, **(B)** GSE108474, **(C)** GSE43289, **(D)** TCGA, **(E)** CGGA_325 and **(F)** CGGA_693datasets. **(G-I)** The forest diagrams represent the multi-factor COX regression analysis, in which the variables include TSPAN7, gender, age, pathological grade, IDH mutation status and MGMT methylation status in TCGA **(G)**, CGGA_325 **(H)**, CGGA_693 **(I)**.

The above analysis of the expression pattern of TSPAN7 shows that the expression level of TSPAN7 is correlated with the malignant degree of glioma. To further confirm its reliability, we explored the relationship between TSPAN7 expression and the prognosis of glioma patients in various public datasets. Patients were divided into high and low expression groups according to the median expression of TSPAN7, and the results showed that patients with high TSPAN7 expression had significantly better survival than those with low TSPAN7 expression in different cohorts (GSE16011 HR = 0.56, p < 0.001; GSE108474 HR = 0.62, p < 0.001; GSE43289 HR = 0.64, p = 0.132; CGGA_325 HR = 0.25, p < 0.001; CGGA_693 HR = 0.42, p < 0.001; TCGA HR = 0.3, p < 0.001) (**Figures 3A–F**). Besides, we also performed survival analysis in glioma with different IDH mutant status in TCGA, CGGA and GSE16011 datasets (**Supplementary Figures S4A–H**). Interestingly, all the datasets showed that the high expression of TSPAN7 were associated with better prognosis except TCGA IDH wildtype group. Although the p value of it in TCGA IDH wildtype group was not significant, the trend of it was consistent with other cohorts.

Next, multivariate COX regression model were performed to analyze independent prognostic effect of TSPAN7 and clinicopathological factors, such as gender, age, WHO grade, IDH mutation status and MGMT methylation status. Adjusting for other variables, COX regression analysis showed that the expression level of TSPAN7 could be used as an independent prognostic factor for overall survival in TCGA (HR = 0.65, p < 0.05), similar result in CGGA_325 (HR = 0.38, p < 0.001) and CGGA_693 (HR = 0.58, p < 0.01) (**Figure 3G–I**). Therefore, we speculate that TSPAN7 is of great significance for predicting the prognosis of glioma patients.

### 4 Gene function annotation and enrichment analysis revealed the inhibiting function of TSPAN7 in glioma

**Figure 4:**
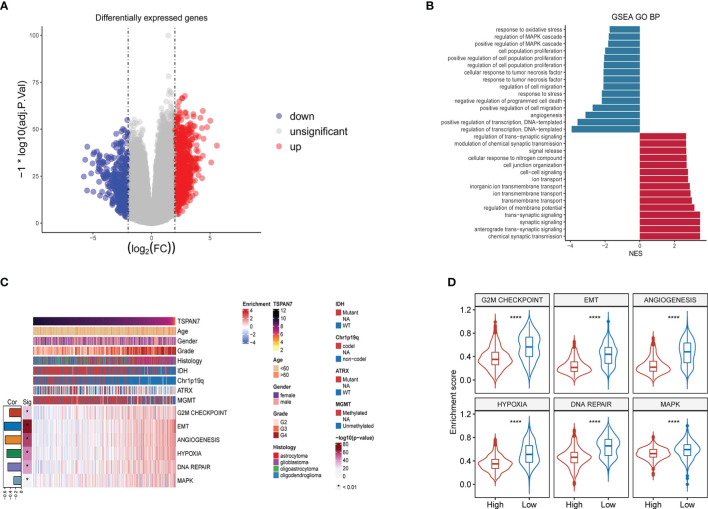
Functional enrichment analysis between the TSPAN7 high- and low‐expression groups in gliomas. **(A)** Differential expression genes (DEGs). **(B)** GO analysis of the up-regulated gene in the high TSPAN7 subgroup and the down-regulated gene in the high TSPAN7 subgroup. **(C)** The relationship between TSPAN7 and proliferation, migration, DNA repair and MAPK signal pathway in the TCGA datasets. **(D)** Differences in enrichment scores between TSPAN7 high and low expression subgroups in cell proliferation, EMT, angiogenesis, hypoxia, DNA repair and MAPK signaling pathways. ****p <.0001.

To further clarify the biological functions that TSPAN7 may be involved during glioma progression, we divided the patients into two groups according to the median expression of TSPAN7 in TCGA glioma cohorts and GO enrichment analysis were performed. By setting (|log fold change (FC)| > 2 and FDR < 0.05) as criteria, we identified 1460 differentially expressed genes (DEGs), including 925 upregulated and 535 downregulated genes in TSPAN7 high expression group (**Figure 4A**). The result of gene set enrichment analysis suggested that downregulated genes were mainly enriched in response to oxidative stress, regulation of MAPK cascade, positive regulation of MAPK cascade, regulation of cell population proliferation, regulation of cell migration, response to stress, and angiogenesis which were related with tumor progression (**Figure 4B**). While upregulated genes were mainly enriched in regulation of trans-synaptic signaling, modulation of trans-synaptic transmission, cellular response to nitrogen compound, cell junction organization, cell-cell signaling, inorganic ion transmembrane transport, regulation of membrane potential and anterograde trans-synaptic signaling which were associated with the neural glial cell development in tumor initiation stage consistent with that TSPAN7 were highly expressed in LGG (27).

Due to the association between abnormal expression of TSPAN7 and some tumor progressed system, we selected some related hallmark gene sets from MSigDB and performed ssGSEA to calculate the activity of each pathway in TCGA cohorts. Interestingly, with the expression levels of TSPAN7 gradually decreasing, the tumor became more malignant and the corresponding biological behaviors, such as cell proliferation capacity, EMT, angiogenesis, DNA repair, as well as MAPK pathways, were more active (**Figure 4C**). Statistical analysis also showed that the enrichment score of each pathway was significantly altered in TSPAN7 low expression subgroup and high expression subgroup (**Figure 4D**). On the basis of these results above, we could infer that high expression of TSPAN7 could inhibit glioma progression.

### 5 Overexpression of TSPAN7 in glioma inhibit Cell Proliferation, Viability, and Migration *In Vitro*

**Figure 5:**
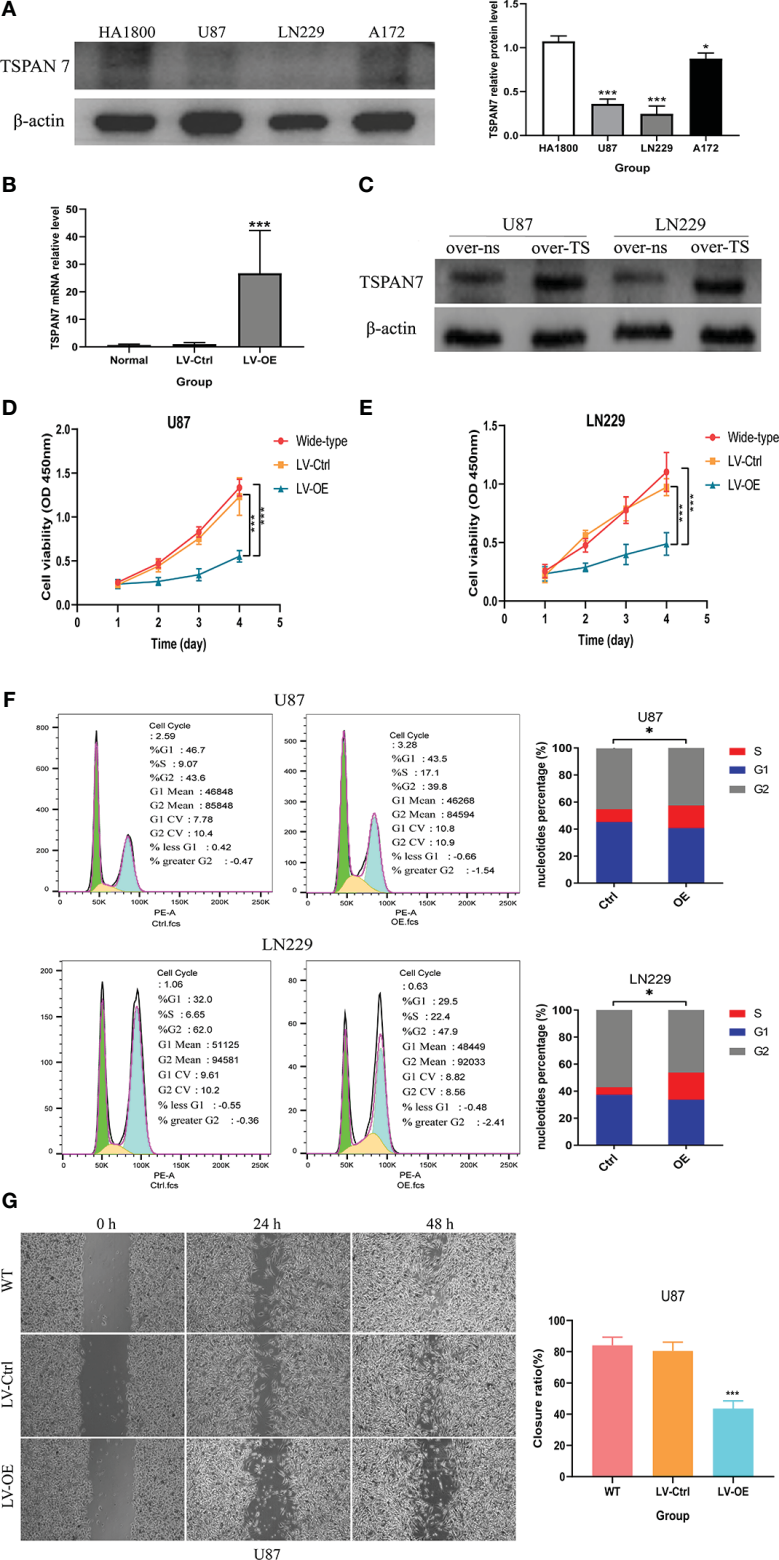
TSPAN7 expression in GBM is protective against cell proliferation, viability and migration in vitro. **(A)** The expression level of TSPAN7 in the three glioma cell lines and normal glia cells were detected by Western blot. **(B, C)** The mRNA expression levels of TSPAN7 in normal, control and TSPAN7-overexpressing cell plasmid transfection were detected by qPCR and Western blot. **(D, E)** CCK-8 assays showed the difference of proliferation capacity between wildtype, LV-Ctrl and TSPAN7-overexpression in U87 and LN229 cell lines. **(F)** Cell cycle differences between TSPAN7-overexpression and control subgroup by flow cytometry in U87 and LN229 cell lines. **(G)** Wound-healing assays showed that cell migration ability between wildtype, LV-Ctrl and TSPAN7 overexpression in U87 cell line, *p <.05, ***p <.001, ns: no statistics.

Through the above bioinformatics analysis, as well as the detection results in clinical glioma samples, we found that TSPAN7 gradually decreased with increasing tumor grade, and higher TSPAN7 expression was associated with a better prognosis. Therefore, we further evaluated whether changes in TSPAN7 expression have an influence on the malignant biological behavior of glioma cells. First, we analyzed TSPAN7 expression in normal glial cell line and glioma cell lines by Western Blotting and found that it was expressed at higher levels in HA1800 than in glioma cell lines (**Figure 5A**). Additionally, TSPAN7 was expressed at relatively low levels in U87 and LN229 cell lines compared with A172. So, we conducted overexpression of TSPAN7 in U87 and LN229. qRT-PCR (**Figure 5B**) and Western Blotting (**Figures 5C**) data confirmed that the expression of TSPAN7 was upregulated in LV-TSPAN7-OE group compared with LV-TSPAN7-Ctrl group. We found that overexpression of TSPAN7 inhibited cell proliferation in U87 and LN229 cell lines (**Figures 5D, E**). In addition, we explored the effect of TSPAN7 on the cell cycle using flow cytometry in U87 and LN229 cell lines, which showed a significantly higher proportion of cells in S-phase in LV-OE TSPAN7 groups compared with LV-Ctrl (**Figure 5F**). Wound-healing assays also demonstrated that over-expression TSPAN7 could inhibited U87 glioma cell migration (**Figure 5G**).

### 6 Relationships between TSPAN7 expression and immune infiltration in glioma microenvironment

**Figure 6:**
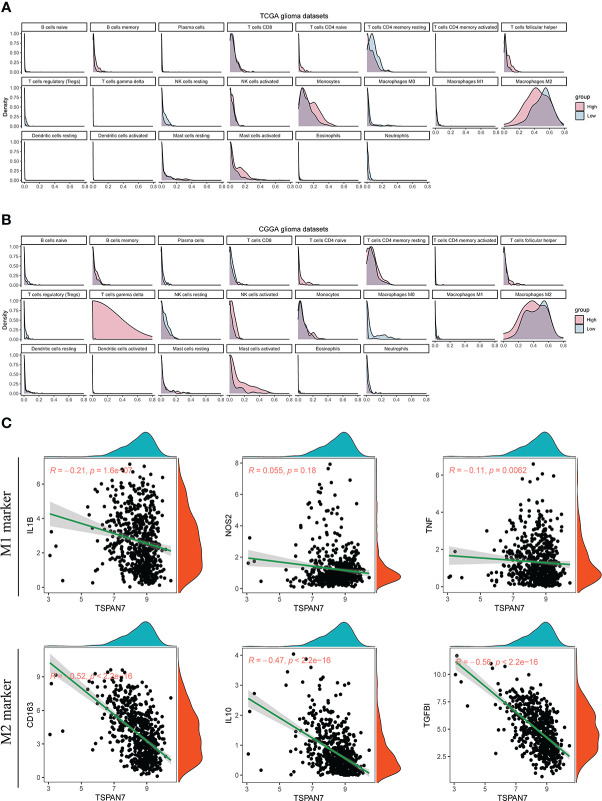
The relationships between TSPAN7 expression and immune infiltration of 22 immune cells in glioma immune microenvironment. **(A, B)** Relative immune cell infiltration level of 22 immune cells between TSPAN7 high expression and low expression subgroup in TCGA and CGGA datasets. **(C)** Correlation analysis of TSPAN7 with M1 type and M2 type macrophage related specific markers in TCGA dataset.

Low expression of TSPAN7 were also associated with immune response (**Supplementary Table S2**) which could influence the tumor-immune cell interaction. To address potential mechanisms underlying these, we used the CIBERSORT algorithm to analyze the relationship between TSPAN7 expression and immune cell infiltrations, including B cells, T cells, natural killer cells, macrophages, dendritic cells, eosinophils, and neutrophils. Results in TCGA dataset showed that tumor with low TSPAN7 expression had dominantly more M2 macrophages infiltrated compared to other immune cells (**Figure 6A**, **Supplementary Figures S5A**). Similar results were found in CGGA datasets (**Figure 6B**, **Supplementary Figures S5B**). Then we examined whether TSPAN7 was associated with the macrophage polarization. Correlation analysis revealed that there was a remarkably negative correlation between TSPAN7 and M2 macrophages markers, such as CD163 (R = - 0.52, p < 2.2×10^-16^)、IL10 (R = - 0.47, p < 2.2×10^-16^) and TGFBI (R = -0.56, p < 2.2×10^-16^). Whereas M1 macrophages markers showed a moderately negative correlation with TSPAN7, such as IL1B (R = - 0.21, p = 1.6×10^-7^), NOS2 (R = - 0.055, p = 0.18) and TNF (R = - 0.11, p = 0.0062) (**Figure 6C**). These results suggested that low TSPAN7 expression may affect the polarization of macrophages and promote an immunosuppressive microenvironment in glioma.

### 7 Evaluation of Response to Immunotherapy Based on the expression of TSPAN7 in glioma

**Figure 7:**
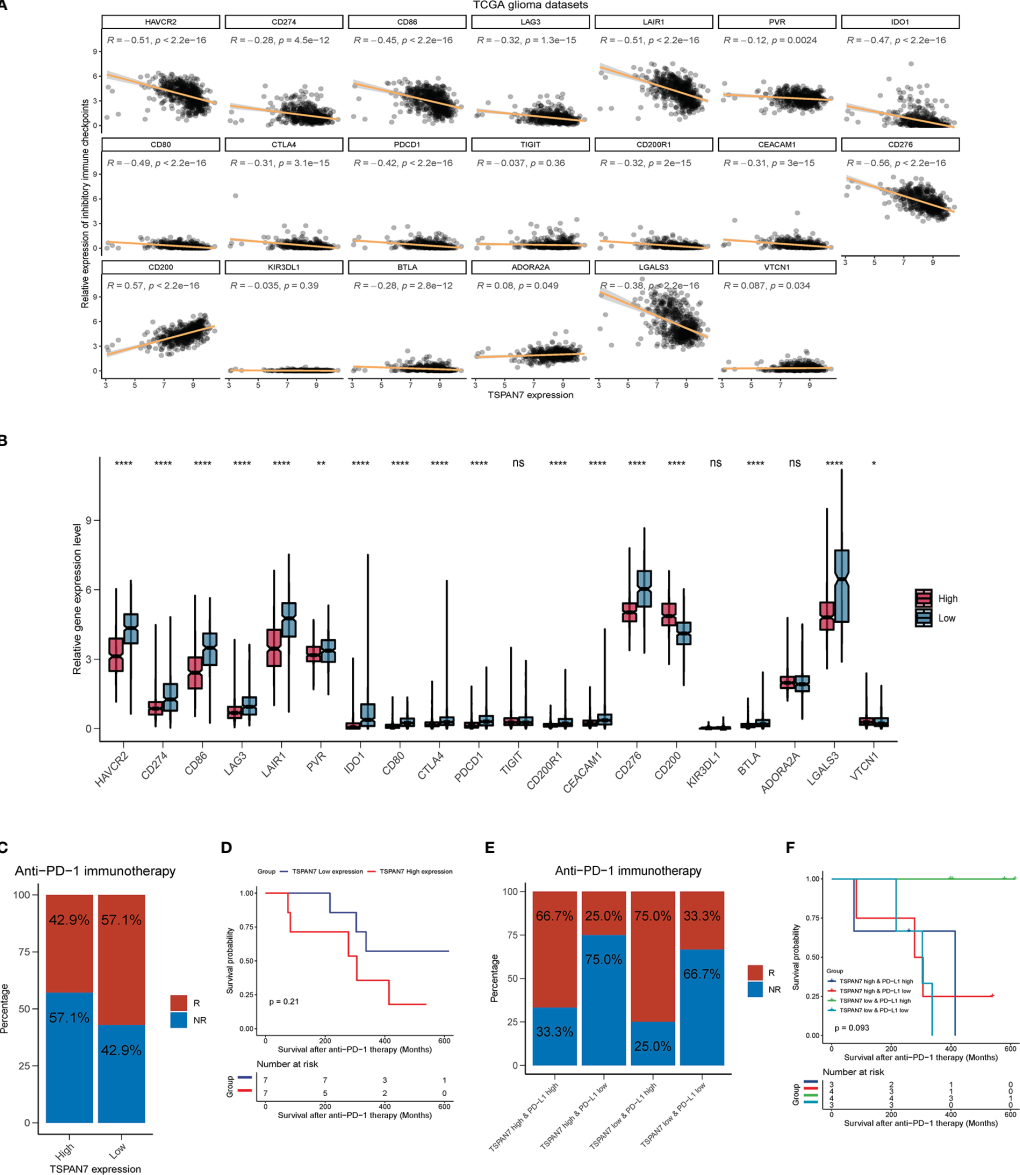
Low TSPAN7 patients are more practical to anti-PD-1 immunotherapy. **(A)** Correlation analysis of TSPAN7 expression with immune checkpoint markers in TCGA dataset. **(B)** Relative expression level differences of different immune checkpoints between TSPAN7 high expression and low expression subgroup in TCGA dataset. **(C, D)** Differences in the proportion of patient treatment response to anti-PD-1 immunotherapy and survival differences between TSPAN7 high and low expression subgroups. **(E, F)** Differences in the proportion of patient treatment response to anti-PD-1 immunotherapy and survival differences between TSPAN7 high or low expression subgroups combined with PDL1 high or low expression. *p <.05, **p <.01, ****p <.0001, ns, no statistics.

As TSPAN7 was associated with immunosuppressive microenvironment, we further assessed the relationship between TSPAN7 expression and immune checkpoints, and found that TSPNA7 expression was negatively correlated with PDCD1 (R = - 0.42, p < 2.2 × 10^-16^), CD274 (PDL1) (R = - 0.28, p = 4.5 × 10^-12^), CTLA4 (R = - 0.31, p = 3.1 × 10^-15^), HAVCR2 (R = - 0.51, p < 2.2 × 10^-16^), CD86 (R = - 0.45, p < 2.2 × 10^-16^), CD276 (R = - 0.56, p < 2.2 × 10^-16^) and LGALS3 (R = - 0.38, p < 2.2 × 10^-16^) in TCGA dataset (**Figure 7A**). Also dividing the patients into high and low expression groups, statistical analysis revealed that PDCD1, CD274 (PDL1), CTLA4, HAVCR2, CD86, CD276 and LGALS3 were significantly higher in the TSPAN7 low expression group compared to the TSPAN7 high expression group (**Figure 7B**). Similar results were also obtained when analyzing CGGA dataset (**Supplementary Figures S6A, B**) indicating that TSPAN7 may play an important role in responding to immunotherapy.

Anti-PD1 or PD-L1 immunotherapy are ongoing in many clinical traits including GBM. Zhao J, et al. recently profiled an GBM cohort with PD-1 inhibitors treatment to understand the molecular determinants of immunotherapeutic response (28). In these cohort, GBM patients with high PD-L1 expression were more possible to benefit from anti-PD-1 immunotherapy and had better prognosis (**Supplementary Figures S6C, D**). In the same cohort, the proportion of clinical response to anti-PD-1 immunotherapy was also higher in the TSPAN7 low expression group (**Figure 7C**), and survival analysis showed that after receiving anti-PD-1 therapy, there was a dominant trend for better prognosis in the TSPAN7 low expression group than in the TSPAN7 high expression group (**Figure 7D**). These results suggested that TSPAN7 expression may had a synergistic effect with PD-L1 on the response to immunotherapy. So, we divided patients into four group based on the expression of TSPAN7 and PD-L1. Interestingly, the proportion of patients responding to treatment was the highest in TSPAN7 low & PD-L1 high expression group which was higher than separate classification based on PD-L1(**Figure 7E**). The results of survival analysis also showed that patients with TSPAN7 low & PD-L1 high expression were almost all alive in follow-up time compared to other groups, which were also more effective than separate classification based on PD-L1(**Figure 7F**). With respect to the above results, TSPAN7 expression not only has link with immune infiltration but also provides a potential value for predicting treatment response to immunotherapy for glioma.

## Discussion

GBM is one of the most aggressive cancers, and there is no effective treatment at present. Although the development of chemotherapeutic drugs has made progress, the overall survival after GBM diagnosis is still not ideal due to the existence of intra- and inter-tumoral heterogeneity. Therefore, novel therapeutic approaches are urgently needed to be explored for treating gliomas.

The tetraspanins is a family of transmembrane proteins, structurally related to voltage dependent Ca2^+^ channels γ Subunits, and can regulate many biological processes including calcium ion influx (29). Among them, the expression of TSPAN7 vary in different tissues, with the highest level in brain tissue, and multiple mutations in TSPAN7 have been implicated in intellectual disability. Lee et al. found that reducing TSPAN7 expression on the cell surface by participating in the internalization process of the inhibitory neuronal DRD2 receptor is closely related to psychiatric disorders such as schizophrenia and is a target of antipsychotics commonly used in clinical practice (30). In the study by Bassani et al, TSPAN7 over-expression can promote the formation of filopodia and dendritic spines, and knockdown of TSPAN7 can cause alterations in the size of spine heads, suggesting that TSPAN7 is indispensable for the stability of spines and normal synaptic transmission (13). Another possible mechanism speculated for TSPAN7 function is *via* PI4k and/or β 1-integrin to influence actin filaments and in turn regulate cytoskeletal architecture (31, 32). Kwon et al. also showed that TSPAN7 disrupts the microtubule network and causes cytoskeletal abnormalities by modulating Src, Pyk2, and microtubule signaling in the process of osteoclast differentiation and functional maturation (33). Usardi et al. showed that, in mouse cerebellar granule cells, TSPAN7 can promote axonal branching and increase the size of TSPAN7 clusters by downregulating IGSF3 expression, which considered as a critical process during brain development (34). In recent years, Ma et al. found that cells continually release contents to the extracellular space during migration and leave their contractile fibers on the side of the soma, where they would form numerous pomegranate-like structures and named ‘migrasome’ (35). TSPANs family members, especially TSPAN4 and TSPAN7, play an important role in the process of migrasome formation (36–38). However, the function and expression characteristics of TSPAN7 have never been reported in glioma.

In the present study, our results indicated that TSPAN7 may have an anti-tumor effect on glioma. We utilized multiple public databases to analyze the function of TSPAN7 in glioma and found that TSPAN7 was significantly decreased in GBM compared with LGG. These results were validated in our clinical samples. Moreover, low expression of TSPAN7 was highly expressed in IDH wildtype, MGMT unmethylated and MES-like tumor. Both survival analysis and multivariate Cox regression analysis in multiple datasets revealed that low TSPAN7 was an independent predictor of prognosis in glioma patients. All these results indicated that TSPAN7 expression was closely related to the malignancy and prognosis of glioma.

To reveal underlying mechanism of TSPAN7 in glioma, we performed enrichment analysis and found that low expression of TSPAN7 was associated with response to oxidative stress, regulation of MAPK, cell population of proliferation, response to tumor necrosis factor, regulation of cell migration, negative regulation of programmed cell death and angiogenesis, which were consistent with our cell functional experiments. Previously, Dickerson et al. have presented compelling evidence indicating that TSPAN7 is a new regulatory subunit of Cav channels, with an important role in Ca2^+^ handling and GSIS in pancreatic β-cells (39). TSPAN7-KD enhanced glucose-stimulated Ca2^+^ influx in both mouse and human β-cells, and increased depolarization-stimulated Ca2^+^ influx in human β-cells. Combined with the findings of this study, we speculate that, during the malignant progression of glioma, with the gradual decrease of TSPAN7, Ca2^+^ influx increased, and the biological behavior such as oxidative stress, cell proliferation, and migration of tumor cells gradually increase.

High grade glioma progresses rapidly, resulting in short survival time of patients. Previous study has confirmed that the level of immune infiltration and activation of immune cells in TIME will contribute notably to progress of tumor and prognosis of glioma patients (40, 41). For example, inflammatory responses can promote tumorigenesis. Tumor associated inflammatory responses can mediate a large number of suppressor cytokines releasing in the tumor microenvironment and recruiting macrophages, neutrophils, regulatory T cells (Tregs) as well as cells myeloid-derived suppressor cells (MDSCs) to inhibit immune activity. Clinical studies have found that the level of macrophage infiltration in the immune microenvironment is associated with the prognosis of glioma patients (42, 43). Tumor associated macrophages can suppress the immune system through multiple pathways, which can be divided into M1 macrophages and M2 macrophages. As tumor progresses, the constitution of immune cells in microenvironment changes, especially the proportion of M2 macrophages increases, so that the immune cells not only have anti-tumor effects, but also promote the immune escape of tumor cells and then contribute to tumor growth (44, 45). Besides, M2 macrophages mainly participate in Th2 immune response and contribute to tumor progression (46, 47). Interestingly, based on the above bioinformatic analysis, we found that the expression level of TSPAN7 was negatively correlated with tumor associated macrophage infiltration, especially M2 subtype macrophage. The possible explanation is that, with the gradually decrease of TSPAN7 expression, tumor cells will promote the recruitment of macrophages and mediate the polarization of M1 macrophages to M2 macrophages, and thus remodeling TIME and inhibiting anti-tumor immune response. Besides, in analysis of relationship between TSPAN7 expression and 22 immune cells, we could find that high TSPAN7 expression were all associated with high CD4 T cell and low Tregs infiltration in TCGA and CGGA datasets (**Figure 6** and **Supplementary Figure S5**). While CD4^+^ T cells primarily mediated anti-tumor immunity and Tregs primarily mediated pro-tumour immunity, these were consistent with our previous result that patients with high TSPAN7 expression had better prognosis.

Immune checkpoints refer to a class of molecules between T cells and antigen-presenting cells that potently inhibit T cell function and, in turn, limit immune responses (48). Immune checkpoint blockade (ICB) therapies are a class of therapeutic approaches that boost anti-tumor immune responses by modulating T cell activity to block inhibitory signals and promote antitumor effects (49). Although immune system can recognize malignant tumor cells, the function of anti-tumor T cell is reduced due to the upregulation of inhibitory immune checkpoints in the TIME, which in turn leads to the failure of the immune response against cancer cells. So, blocking or inhibiting immune checkpoints, enhancing anti-tumor immune responses, provides new ideas for the clinical treatment of malignant tumors. Because PD1 and CTLA4 are commonly expressed on the surface of activated T cells, PD-1 monoclonal antibody and CTLA-4 monoclonal antibody could block inhibitory signaling from tumor cells (50, 51). In addition, immunosuppressive molecules such as PD-L1, TIGIT and CD80, which are expressed at increased levels on glioma cells, also negatively regulate the interaction between antigen-presenting cells and T cells (52–54). In this study, we found that TSPAN7 expression was negatively associated with several immune checkpoints related genes, such as PDCD1, CTLA4, HAVCR2, CD276, CD80, IDO1 and LGALS3, suggesting that TSPAN7 plays a synergistic role with those immune checkpoints during the progression of GBM. Furthermore, based on the existing datasets of immunotherapy in GBM, we found that patients with low expression of TSPAN7 and high PD-L1 expression had more response to anti-PD-1 immunotherapy, indicating that anti-PD-1 immunotherapy, combined with over-expressing TSPAN7, could improve the outcomes of glioma. But it still needs more clinical traits to confirm these results because of limit number of immunotherapy cohorts in GBM up to now. Previous study has confirmed that dendritic cell maturation regulates TSPAN7 function in HIV-1 transfer to CD4+ T lymphocytes (55). Besides, during macrophage differentiation into osteoclasts, TSPAN7 is involved in their morphogenesis and required for the formation of the podosome belt (33). Therefore, TSPAN7 appears as a master regulator of morphological changes occurring during cell differentiation through cytoskeleton remodeling. PDL1 is mainly expressed on the surface of tumor cells and antigen-presenting cells (dendritic cells, macrophages, etc.). The up-regulation of PDL1 expression on the surface of tumor cells will promote the immune escape of tumor cells. Therefore, we hypothesized that TSPAN7 may affect the binding of PDL1 to PD1 in tumor cells by changing the morphology of tumor cells, and then affect the efficacy of anti-PDL1 immunotherapy.

In summary, our findings initially illustrate that TSPAN7 can be a novel effective indicator for predicting the clinical stage, and has multifaceted prognostic value in glioma. The relationship between TSPAN7 expression and TIME is particularly noteworthy that patients with lower TSPAN7 expression were more likely to benefit from anti-PD-1 immunotherapy. These results are of great significance to clinical management, which will be conducive to the precise treatment of patients with glioma.

## Data availability statement

The data for the bioinformatics analysis in this study were obtained from online database data and are available through: UCSC Xena (https://xenabrowser.net/), CGGA website (http://www.cgga.org.cn/) and GEO datasets. Further inquiries can be directed to the corresponding authors.

## Ethics statement

This study was reviewed and approved by the ethics committee of Xiangya Hospital, Central South University.

## Author contributions

LC designed the project, implemented experiment, analyzed the data and wrote manuscript; HL and XL (corresponding author) conceived the project, analyzed the data and revised the manuscript. YL, XL and SW participated in experiment design and provided a lot of valuable advice. SX collected clinical samples, helped to implement part of experiments and approved the final draft. All authors contributed to the article and approved the submitted version.

## Supplementary material

Supplementary Figure 1 Clinical and molecular characteristics of TSPAN7 for gliomas in TCGA and CGGA datasets. **(A, B)** The expression level of TSPAN7 for different WHO pathological grades in CGGA_325 and CGGA_693 datasets. **(C-D)** The expression of TSPAN7 for different glioma pathological types in CGGA_325 and CGGA_693 datasets. **(E-F)** Expression of TSPAN7 stratified by 1p19q co-deletion and non co deletion in CGGA_325 and CGGA_693 datasets. **(G-H)** Expression of TSPAN7 between IDH mutant and wild type in CGGA_325 and CGGA_693 datasets. **(I-J)** The expression of TSPAN7 in IDH mutation status combined with different pathological grades in CGGA_325 and CGGA_693 datasets. **(K-L)** TSPAN7 expression levels stratified by MGMT methylation status in CGGA_325 and CGGA_693 datasets. *p <.05, **p <.01, ***p <.001, ****p <.0001, ns, no statistics.

Supplementary Figure 2
**(A, B)** The expression of marker genes of each cluster in single cell sequencing data.

Supplementary Figure 3
**(A-C)** The expression pattern of TSPAN7 in the spatial transcriptome.

Supplementary Figure 4 Survival analysis between TSPAN7 low- and high-expression in glioma with different IDH mutant status. **(A-B)** TCGA, TSPAN7 in IDH mutant and Wildtype **(WT)** glioma, **(C-D)** CGGA_325, TSPAN7 in IDH mutant and WT glioma, **(E-F)** CGGA_693, TSPAN7 in IDH mutant and WT glioma, **(G-H)** GSE16011, TSPAN7 in IDH mutant and WT glioma.

Supplementary Figure 5 The relationships between TSPAN7 expression and immune infiltration of 22 immune cells in glioma immune microenvironment. **(A, B)** Relative immune cell infiltration level of 22 immune cells between TSPAN7 high expression and low expression subgroup in **(A)** TCGA and **(B)** CGGA datasets. *p s<.05, **p <.01, ***p <.001, ****p <.0001, ns: no statistics.

Supplementary Figure 6 Relationships between TSPAN7 expression and immune checkpoint markers. **(A)** Correlation analysis of TSPAN7 expression with immune checkpoint markers in CGGA dataset. **(B)** Relative expression level differences of different immune checkpoints between TSPAN7 high expression and low expression subgroup in CGGA dataset. **(C, D)** Differences in the proportion of patient treatment response to anti-PD-1 immunotherapy and survival differences between PDL1 high and low expression subgroups.

Supplementary Table 1 Clinical characteristics of glioma samples from Xiangya hospital.

Supplementary Table 2 GO enrichment of downregulated genes about immune response.
