# Supplementary figures and images for: Functional characterization of TSPAN7 as a novel indicator for immunotherapy in glioma
Source: Front Immunol. 2023 Feb 9;14:1105489. doi: 10.3389/fimmu.2023.1105489 (PMC9947846; doi:10.3389/fimmu.2023.1105489)

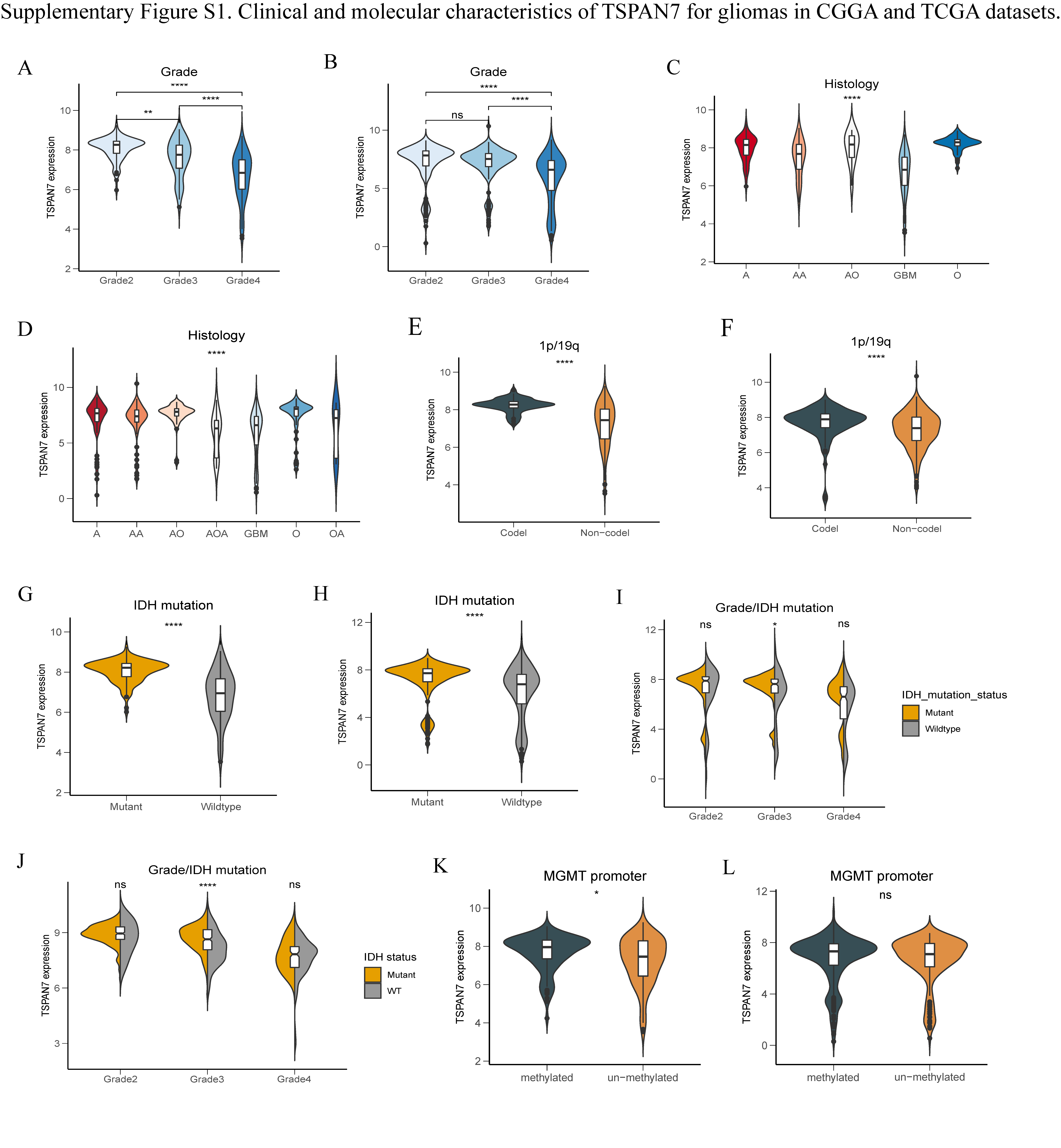

Supplement: Supplementary Figure 1 — Clinical and molecular characteristics of TSPAN7 for gliomas in TCGA and CGGA datasets. (A, B) The expression level of TSPAN7 for different WHO pathological grades in CGGA_325 and CGGA_693 datasets. (C-D) The expression of TSPAN7 for different glioma pathological types in CGGA_325 and CGGA_693 datasets. (E-F) Expression of TSPAN7 stratified by 1p19q co-deletion and non co deletion in CGGA_325 and CGGA_693 datasets. (G-H) Expression of TSPAN7 between IDH mutant and wild type in CGGA_325 and CGGA_693 datasets. (I-J) The expression of TSPAN7 in IDH mutation status combined with different pathological grades in CGGA_325 and CGGA_693 datasets. (K-L) TSPAN7 expression levels stratified by MGMT methylation status in CGGA_325 and CGGA_693 datasets. *p <.05, **p <.01, ***p <.001, ****p <.0001, ns, no statistics. [file Image_1.tif]

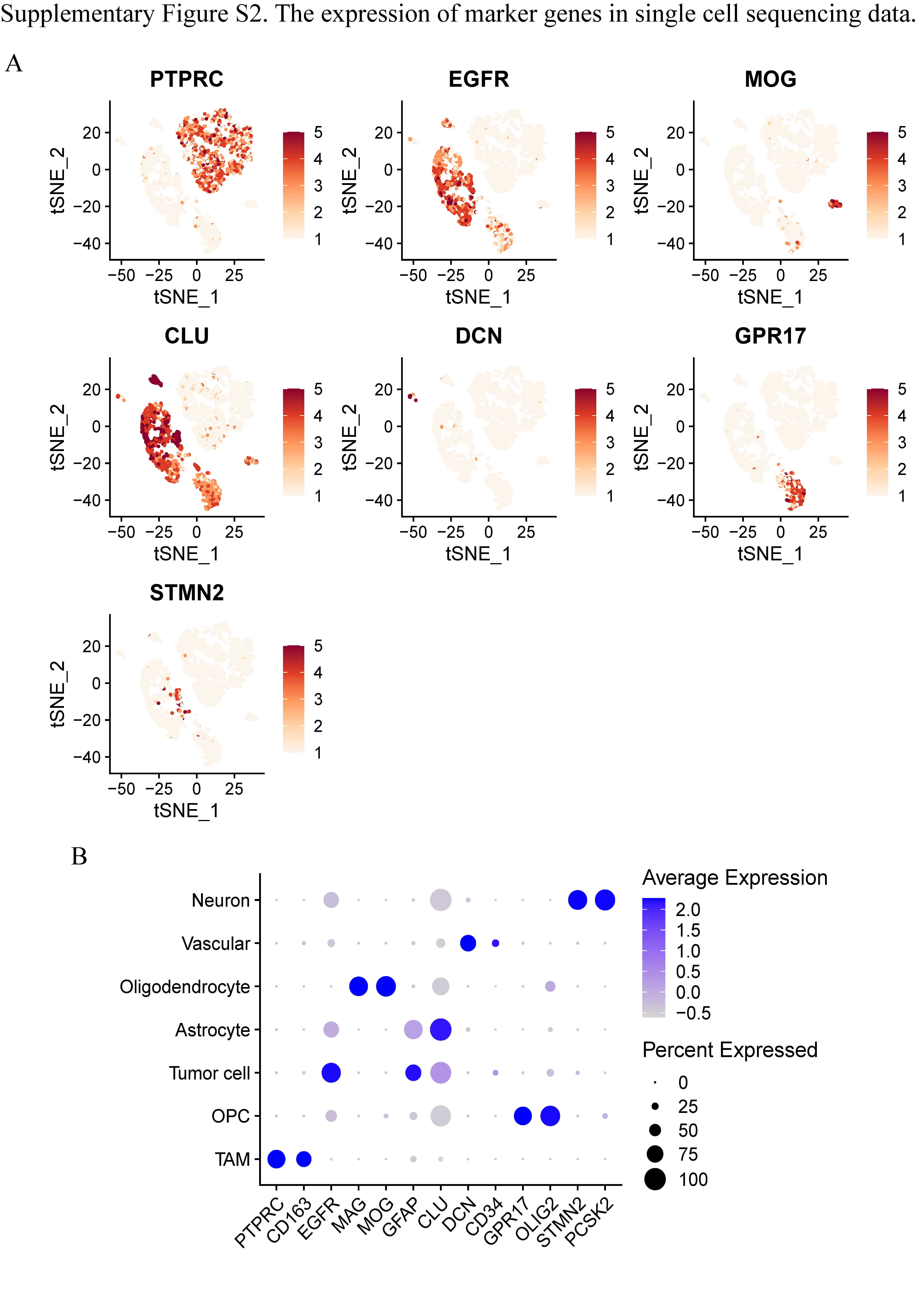

Supplement: Supplementary Figure 2 — (A, B) The expression of marker genes of each cluster in single cell sequencing data. [file Image_2.tif]

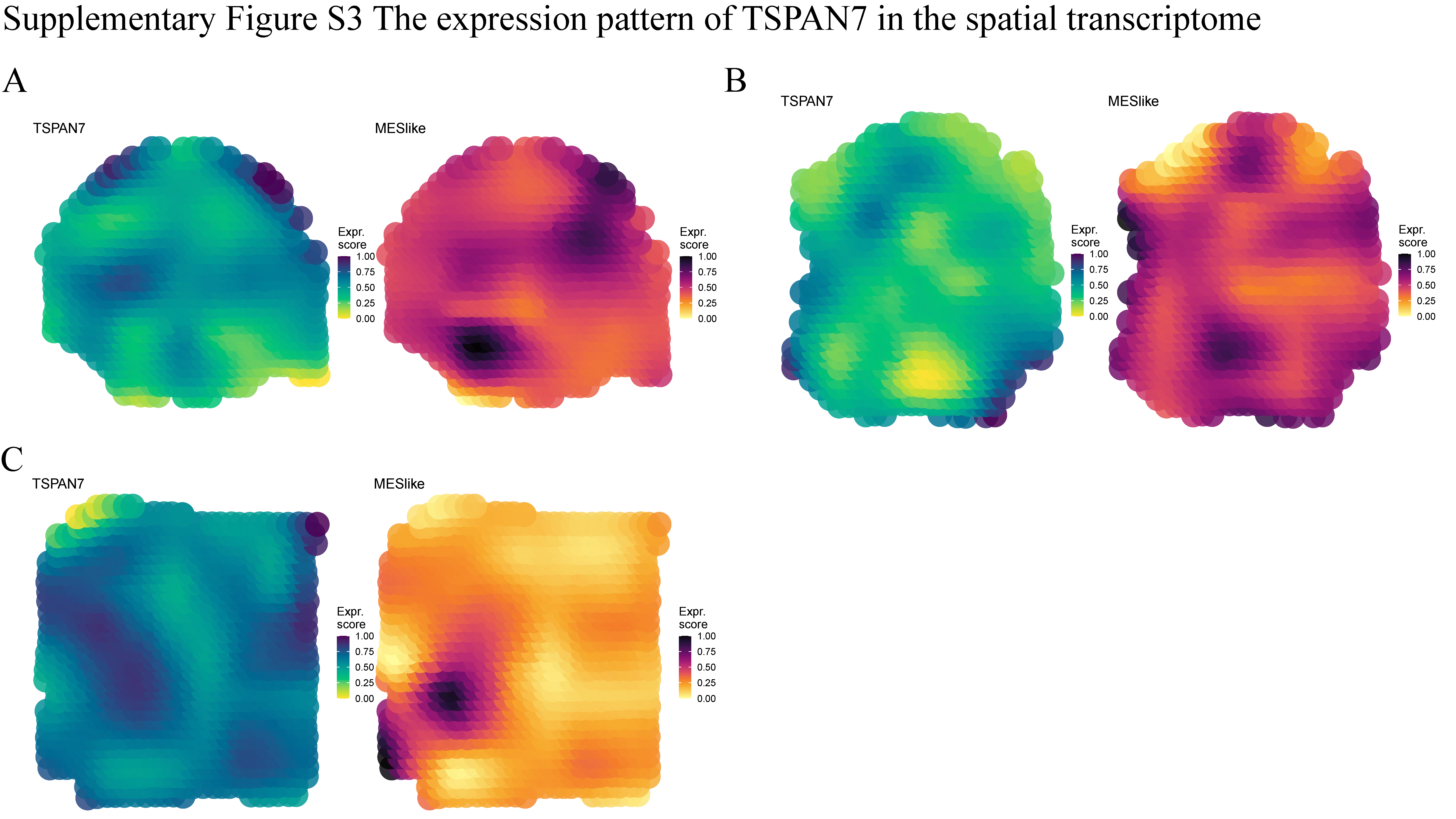

Supplement: Supplementary Figure 3 — (A-C) The expression pattern of TSPAN7 in the spatial transcriptome. [file Image_3.tif]

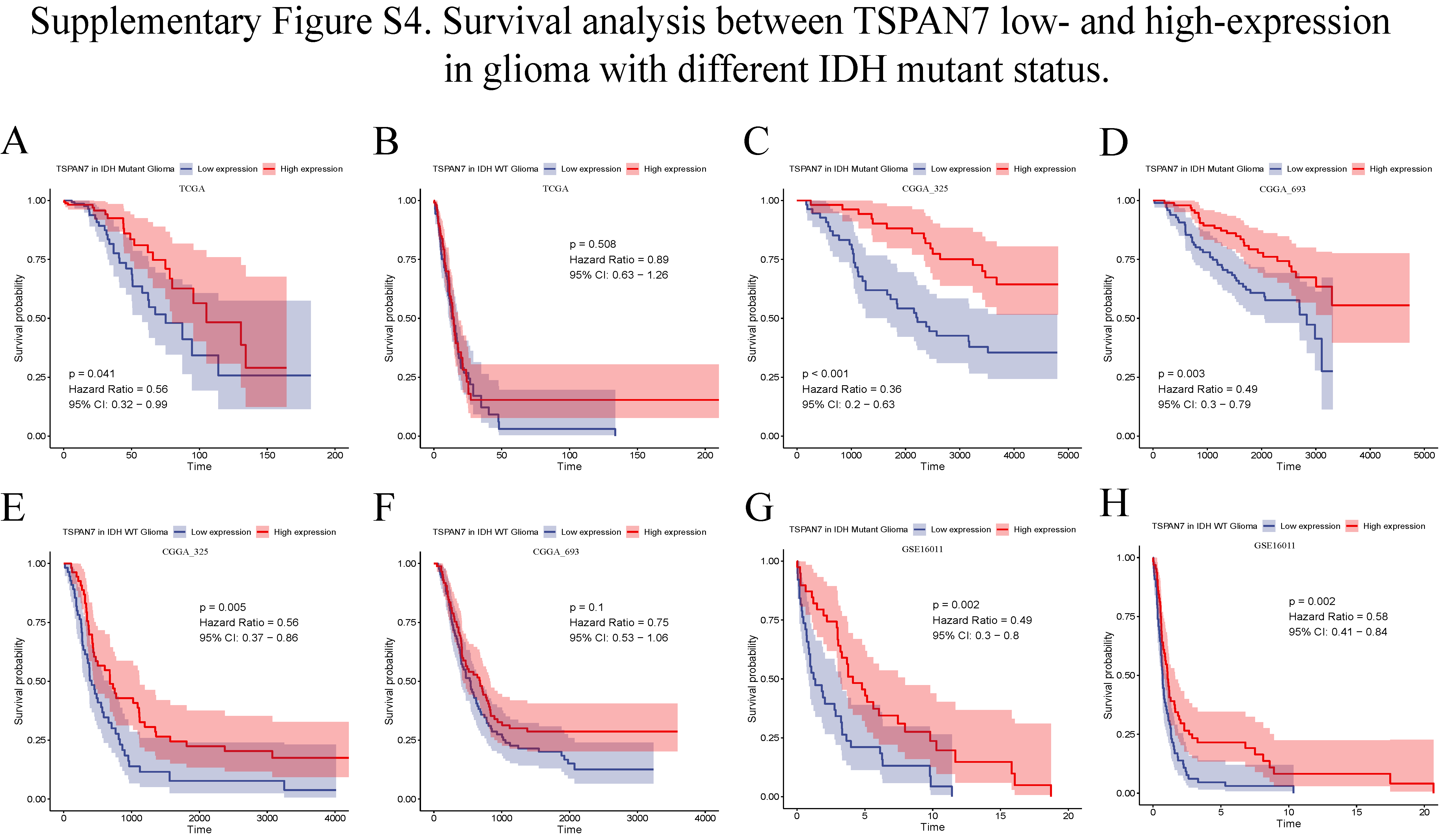

Supplement: Supplementary Figure 4 — Survival analysis between TSPAN7 low- and high-expression in glioma with different IDH mutant status. (A-B) TCGA, TSPAN7 in IDH mutant and Wildtype (WT) glioma, (C-D) CGGA_325, TSPAN7 in IDH mutant and WT glioma, (E-F) CGGA_693, TSPAN7 in IDH mutant and WT glioma, (G-H) GSE16011, TSPAN7 in IDH mutant and WT glioma. [file Image_4.tif]

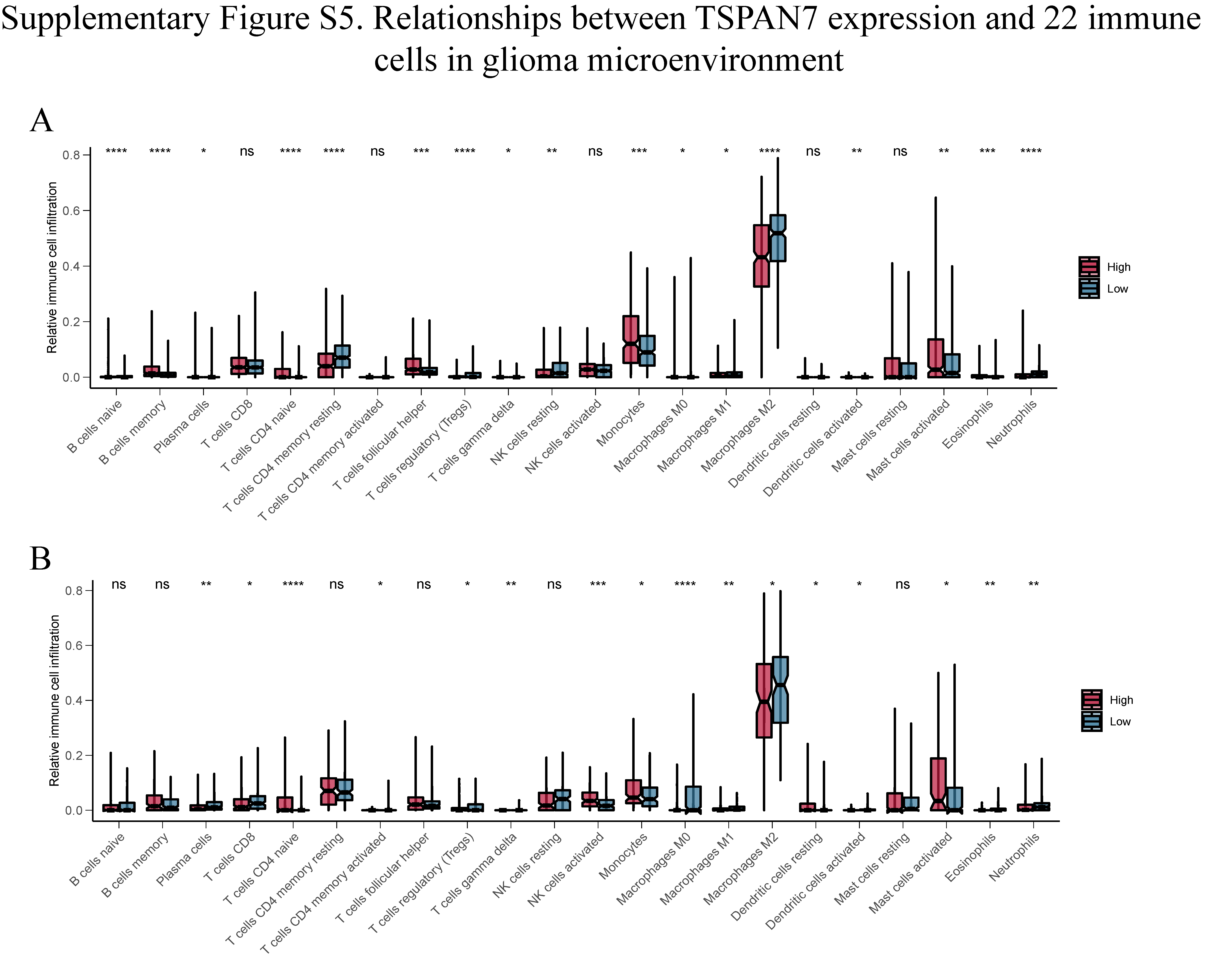

Supplement: Supplementary Figure 5 — The relationships between TSPAN7 expression and immune infiltration of 22 immune cells in glioma immune microenvironment. (A, B) Relative immune cell infiltration level of 22 immune cells between TSPAN7 high expression and low expression subgroup in (A) TCGA and (B) CGGA datasets. *p s<.05, **p <.01, ***p <.001, ****p <.0001, ns: no statistics. [file Image_5.tif]

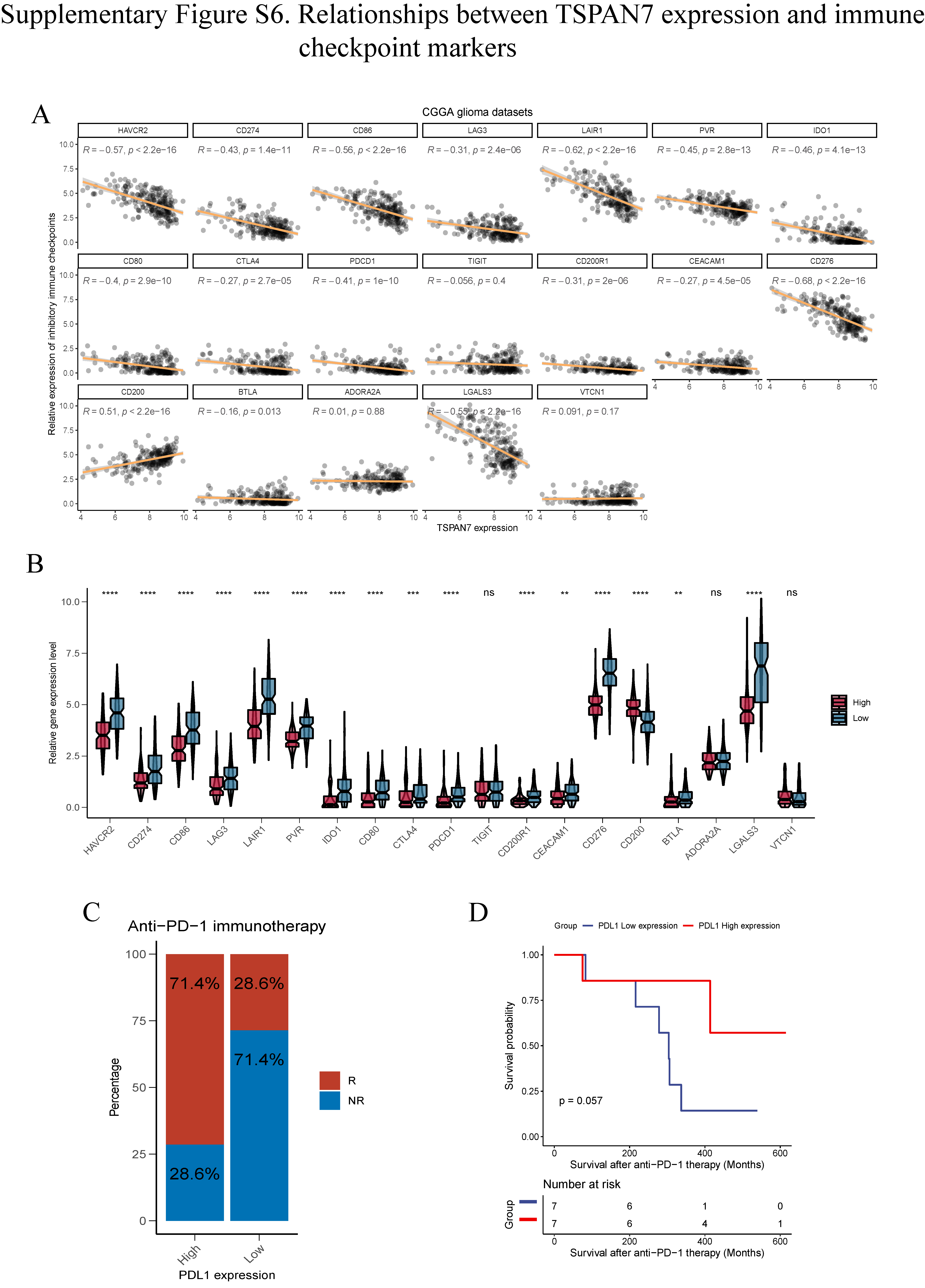

Supplement: Supplementary Figure 6 — Relationships between TSPAN7 expression and immune checkpoint markers. (A) Correlation analysis of TSPAN7 expression with immune checkpoint markers in CGGA dataset. (B) Relative expression level differences of different immune checkpoints between TSPAN7 high expression and low expression subgroup in CGGA dataset. (C, D) Differences in the proportion of patient treatment response to anti-PD-1 immunotherapy and survival differences between PDL1 high and low expression subgroups. [file Image_6.tif]
